# The wealth gradient in diarrhoea, acute respiratory infections, and malaria in childhood over time: A descriptive analysis using DHS and MICS from Western and Central Africa between 1995 and 2017

**DOI:** 10.7189/jogh.11.13009

**Published:** 2021-08-10

**Authors:** Ann-Charline Weber, Lisa Bogler, Sebastian Vollmer, Aline Simen-Kapeu, Rene Ehounou Ekpini, Noel Marie Zagre

**Affiliations:** 1 United Nations Children’s Fund (UNICEF), West and Central Africa Regional Office, Dakar, Senegal; 2 Department of Economics and Centre for Modern Indian Studies, University of Göttingen, Göttingen, Germany; 3 UNICEF Area Representative for Gabon and São Tomé and Príncipe and to the ECCAS, Libreville, Gabon

## Abstract

**Background:**

While the prevalence of childhood diseases and related mortality have been decreasing over the past decades, progress has been unequally distributed. The poorest households often carry the highest disease burden. As morbidity and mortality also decline most slowly among children of the poorest households, socioeconomic status may become a more relevant risk factor for childhood diseases.

**Methods:**

We analysed the association between socioeconomic status and highly prevalent childhood diseases, specifically diarrhoea, acute respiratory infections (ARI), and malaria, and how this association changed over time. For this observational study, we used repeated cross-sectional data, namely all available Demographic and Health Surveys as well as Multi-Indicator Cluster Surveys from Western and Central Africa between 1995 and 2017. We estimated the predicted prevalence of each disease for the entire region in three time periods. We repeated the analysis separately for each country to highlight heterogeneity between countries.

**Results:**

A notable wealth gradient can be seen in the prevalence rates of diarrhoea, ARI, and malaria in Western and Central Africa. Children in the poorest quartile have a much higher morbidity than children in the richest quartile and have experienced a considerably slower decline in prevalence rates. In the period 2010-2017, predicted prevalence of diarrhoea was 17.5% for children in the poorest quartile and 12.5% for children in the richest quartile. Similarly, the predicted prevalence was 11.1% and 8.6% for ARI, and 54.1% and 24.4% for malaria in endemic countries. The pattern does not differ between boys and girls. While exact prevalence rates vary between countries, only few countries have seen a decline in the wealth gradient for childhood diseases.

**Conclusions:**

The increasing wealth gradient in health raises concerns of increasing inequality that goes beyond wealth. It suggests a need to further improve targeting of health programmes. Moreover, these programmes should be adapted to address the interlinked challenges which burden the poorest households.

National as well as global programmes such as the WHO Global Malaria Programme (GMP) and The Global Action Plan for the Prevention of Pneumonia and Diarrhoea (GAPPD) strive towards a reduction in morbidity and mortality due to preventable childhood diseases [1,2]. Over the past decades, the prevalence of childhood diseases and related mortality have in fact been decreasing worldwide [3-5]. While this is in great part due to large-scale interventions such as vaccination programmes and other child health initiatives [5,6], economic development may generally also contribute through changes in the distribution of risk factors. Access to safe water and basic sanitation facilities, for example, has increased in recent years [7], thereby reducing the risk of diarrhoea on the individual level [8]. Similarly, as families move from traditional to modern, improved housing, the risk of malaria is reduced [9].

However, progress is unequally distributed and slowing down [2-4,10]. The poorest households often carry the highest disease burden [11-13]. Marginalised, hard-to-reach communities are underserved by crucial child health interventions and lack access to basic facilities in several countries [7,14]. Depending on which population groups benefit from large-scale health initiatives or general economic development, socioeconomic status may become a more relevant risk factor for childhood diseases. It is therefore important to assess how these risk factors change over time.

Evidence from single countries suggests that children in poorer households are at higher risk of being infected with childhood diseases. Kahabuka et al. found a positive association between risk of severe pneumonia and diarrhoea and lower socioeconomic status in Tanzania [15]. Similarly, wealthier people were found to have a lower risk of malaria infection in the Democratic Republic of Congo [16]. A few studies analyse the association between socioeconomic status and morbidity for country groups. Mfueni Bikundi and Coppieters identified household wealth status as important risk factor for malaria in sub-Saharan Africa [17]. Looking at all low- and middle-income countries, Fuller et al. compared diarrhoea prevalence among children from households that share sanitation facilities with children from households that use non-shared facilities and estimated that a large fraction of the difference in diarrhoea prevalence could be explained by differences in socioeconomic status [18].

We contribute to this evidence by analysing the association of socioeconomic status with highly prevalent childhood diseases, namely diarrhoea, acute respiratory infections (ARI), and malaria, over time. We present the change of the wealth gradient in disease prevalence. Due to limited data on malaria, we also look at fever as a proxy of malaria. Our regional focus is on Western and Central Africa, given that several of the countries with the highest burden of diarrhoea and pneumonia are located in Western and Central Africa, including Nigeria, the Democratic Republic of the Congo, Chad, Niger, and Côte d’Ivoire [6]. Similarly, the two countries with the most malaria cases in 2017 were Nigeria and the Democratic Republic of Congo [2]. Moreover, progress in reducing these prevalences has slowed down in Western and Central Africa over the past decades [10]. We use all Demographic and Health Surveys (DHS) as well as Multi-Indicator Cluster Surveys (MICS) from this region between 1995 and 2017 for which information on these childhood diseases is available.

## METHODS

### Data

For this observational study, we used repeated cross-sectional, nationally representative survey data from Western and Central Africa. We combined two data sources, the DHS and MICS. The DHS is administered by ICF International. In seven rounds since 1984, the DHS Program has collected nationally representative data in low- and middle-income countries. MICS is a data collection initiative by UNICEF and has collected data on women and children in low- and middle-income countries since 1995. In both survey programmes, women in reproductive age, typically between 15 and 49, were asked for information on all children ever born to them. The surveys apply a multistage stratified sampling design for the within-country selection of households. For each country, regions were defined, within which the population was stratified into urban and rural. For each stratified area, enumeration areas were randomly drawn and denoted as primary sampling units (PSUs). Selection of PSUs was based on a probability proportional to size, in this case the number of households. Within each PSU, all households were listed from the most recent population census. Applying an equal probability systematic sampling, a fixed number of households in each PSU was selected for an interview. Weights for the calculation of nationally representative statistics are provided with the survey data. For the analysis across countries, we re-scaled weights using each country’s female population aged 15 to 49 years in the last year of the considered time period. This ensures that small countries do not excessively influence the estimation of overall disease prevalence.

**Figure 1:**
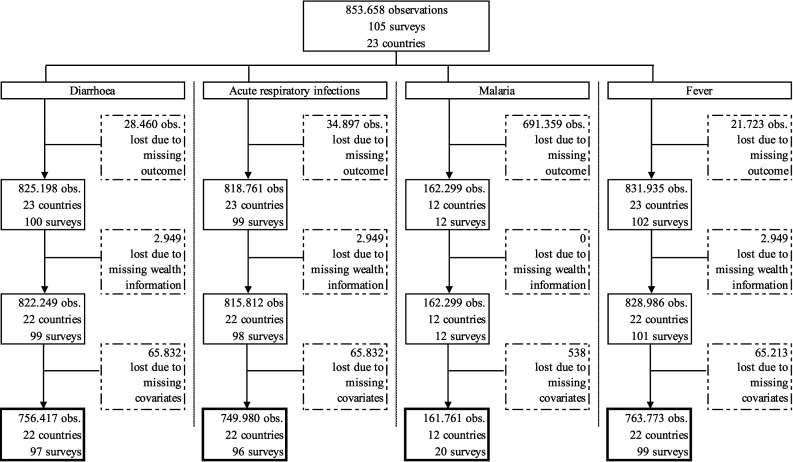
Sample selection.

In this analysis, we included data from 1995 to 2017 for all countries in Western and Central Africa for which data for outcomes, exposure, and covariates was available. Observations with missing information in any of these variables had to be dropped. **Figure 1** presents the sample selection in greater detail.

### Outcomes

We analysed the association of socioeconomic status with three childhood diseases, namely diarrhoea, acute respiratory infections (ARI), and malaria, as well as fever as a proxy for malaria. All diseases were coded as dummy variables that equal one if the child has the disease. A child was defined as having diarrhoea or fever if his/her guardian responded that the child suffered from diarrhoea or fever in the past 24 hours. Malaria was identified by a positive result of the rapid diagnostic test. A child was defined as having ARI if he/she had cough in the past 24 hours and experienced short, rapid breathing. A stricter definition would identify the disease as ARI only if the breathing difficulties were related to a problem in the chest. However, only more recent surveys included the question whether the child’s difficulty in breathing was due to a problem in the chest or a blocked nose, which would not allow for an analysis of the change in the association. We looked at ARI as proxy for pneumonia, despite evidence that it is not a very accurate indicator [19,20]. Unfortunately, the DHS and MICS do not contain more accurate data on pneumonia such as diagnostic tests.

### Exposure and covariates

To capture the socioeconomic status of a child’s household, we created an asset index of observed household assets. While many of the included surveys provided an asset index or a variable identifying the wealth quintile of a household, we preferred to calculate an asset index comparable across countries for as many surveys as possible. Our asset index was calculated using principal-component analysis to define asset-based survey-specific wealth quartiles based on the same list of assets for each survey. Included assets were radio, television, bicycle, car, motorcycle, refrigerator, phone, electricity, piped drinking water, flush toilet, floor material, wall material, and roof material. Households with information on less than 88% of underlying assets were excluded. This cut-off was chosen to include the maximum information about assets while retaining as many country-years as possible. Due to low variation in asset ownership in earlier years for some surveys, few wealth quartiles contain no actual observations.

Covariates used in the analysis for the prediction of disease prevalence, included the child’s gender and age in years, the mother’s age at birth of the child and her educational level (none, primary, or secondary and higher), as well as the location of the household (rural or urban).

### Statistical analysis

In order to assess the change over time in the association between socioeconomic status and disease prevalence, we split the data into three time periods. Time periods were defined such that each country had at least one survey in each time period, as far as possible. This resulted in the earliest period containing surveys between 1995 and 2001, the middle period containing surveys between 2002 and 2009, and the most recent time period containing surveys between 2010 and 2017. Since rapid tests for identifying malaria were only conducted in more recent surveys, associations with malaria prevalence could only be shown for the recent time period.

We estimated the predicted prevalence of each disease for the whole sample in the three time periods, and repeated the analysis separately for each country to highlight heterogeneity between countries.

The predictions were based on regressions of the disease on the set of covariates mentioned above. Furthermore, we added survey year- and country-fixed effects as country level factors have been shown to influence childhood diseases such as diarrhoea [21]. No further sensitivity analyses were performed. All analyses were performed using Stata 14.

### Ethical approval

Ethical approval was not obtained for this study due to the use of secondary data. Both DHS and MICS obtain ethical approval for data collection.

## RESULTS

**Figure 2:**
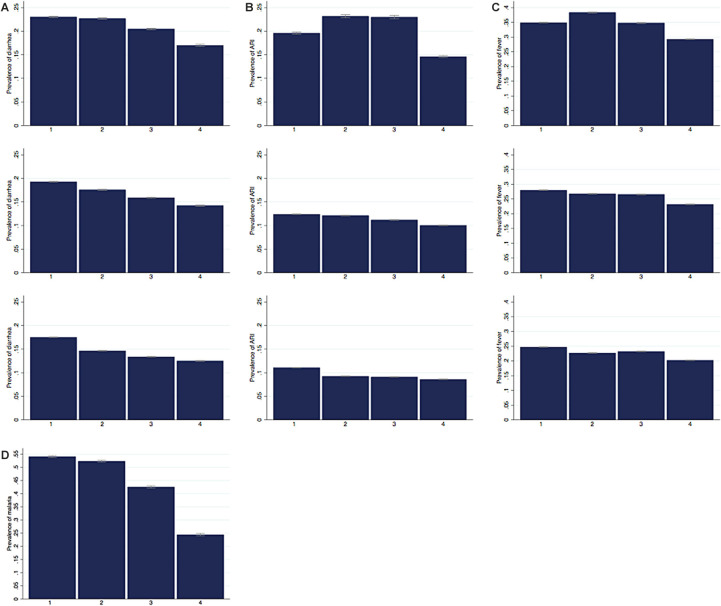
Predicted prevalence of childhood diseases across wealth quartiles over time. **Panel A.** Predicted prevalence of diarrhoea across wealth quartiles over time Note: Predicted prevalence of diarrhoea and 95% confidence intervals across wealth quartiles for all countries in three time periods: 1995-2001, 2002-2009, 2010-2017. Predicted prevalence is calculated separately for each time period from a regression of disease on wealth controlling for child’s gender and age in years, mother’s age at birth of the child, mother’s educational level, location of the household (rural/urban), and survey-year and country-fixed effects. Observation weights are rescaled using each country’s female population aged 15 to 49 years in the last year of the considered time period. **Panel B.** Predicted prevalence of ARI across wealth quartiles over time. Note: Predicted prevalence of ARI and 95% confidence intervals across wealth quartiles for all countries in three time periods: 1995-2001, 2002-2009, 2010-2017. Predicted prevalence is calculated separately for each time period from a regression of disease on wealth controlling for child’s gender and age in years, mother’s age at birth of the child, mother’s educational level, location of the household (rural/urban), and survey-year and country-fixed effects. Observation weights are rescaled using each country’s female population aged 15 to 49 years in the last year of the considered time period. ARI – acute respiratory infections. **Panel C.** Predicted prevalence of fever across wealth quartiles over time. Note: Predicted prevalence of fever and 95% confidence intervals across wealth quartiles for all countries in three time periods: 1995-2001, 2002-2009, 2010-2017. Predicted prevalence is calculated separately for each time period from a regression of disease on wealth controlling for child’s gender and age in years, mother’s age at birth of the child, mother’s educational level, location of the household (rural/urban), and survey-year and country-fixed effects. Observation weights are rescaled using each country’s female population aged 15 to 49 years in the last year of the considered time period. **Panel D.** Predicted prevalence of malaria across wealth quartiles. Note: Predicted prevalence of malaria and 95% confidence intervals across wealth quartiles for all countries, 2010-2017. Predicted prevalence is calculated from a regression of disease on wealth controlling for child’s gender and age in years, mother’s age at birth of the child, mother’s educational level, location of the household (rural/urban), and survey-year and country-fixed effects. Observation weights are rescaled using each country’s female population aged 15 to 49 years in 2017.

**Figure 2**, Panels A-D show the predicted prevalence of diarrhoea, ARI, fever, and malaria for the whole region split by wealth quartiles for three different time periods. Overall, the wealth of the household is associated with the prevalence of the disease. Children from the poorest quartile are more likely to have a disease than children from the richest quartile. The associations differ across diseases and over time.

In the most recent period 2010-2017 the predicted prevalence of diarrhoea decreases with each wealth quartile (**Figure 2**, Panel A). It is 17.5% for children in the poorest quartile, 14.7% and 13.4% for children from the second and third wealth quartile respectively and 12.5% for children from richest quartile. This general pattern also characterises the prevalence in the two earlier time periods. Over time, the predicted prevalence decreased for all quartiles. The decline was relatively larger for the middle quartiles. In the first two periods, the difference between the poorest two quartiles was small with less than one percentage point, but it increased over time to be the largest difference between quartiles with 2.8 percentage points.

The predicted prevalence of ARI is depicted in **Figure 2**, Panel B. While the overall prevalence of ARI is lower compared to the prevalence of diarrhoea, we find a similar picture in the most recent period despite slightly smaller differences between wealth quartiles. The prevalence of ARI is 11.1% for the poorest and 8.6% for the richest quartile. The difference between the second and third quartile is negligible. Interestingly, for the earliest period between 1995 and 2001, the predicted prevalence is highest for the second and the third quartile. The prevalence rates declined remarkably over time for all wealth quartiles, the largest reductions being achieved in the second and third quartile.

For fever, the differences of the predicted prevalence between the wealth quartiles are smaller (**Figure 2**, Panel C). The predicted prevalence is 24.7% for the poorest quartile, and 22.7%, 23.2% and 20.2% for the quartiles two to four respectively. Similar to the pattern observed for ARI, in the earliest period the predicted prevalence is highest for children from the second and third wealth quartile. In all quartiles, the prevalence decreased considerably between the early and the middle time period but only slightly between the middle and the most recent period.

Data on malaria is only available from more recent surveys and for selected countries. We therefore present the predicted prevalence for malaria in the most recent period (**Figure 2**, Panel D). The prevalence rate is high overall. This is in part driven by data availability since rapid malaria tests are only included in the surveys in malaria endemic countries. The difference in malaria prevalence between the richest and poorest quintile is considerable, with 54.1% in the poorest and 24.4% in the richest quartile.

We also analysed whether wealth has a different association with childhood diseases for boys and girls. Figure S1, Panels A-D in the **Online Supplementary Document**, replicates **Figure 2**, Panels A-D, disaggregated by gender. While girls have a slightly lower predicated prevalence of diarrhoea and to a lesser extent of ARI and fever compared to boys, the pattern of the wealth gradient is exactly the same for both genders.

**Tables 1** to **4** show the predicted prevalence by wealth quartile for each country for the first and most recent survey. Overall, the general pattern persists with a lower predicted prevalence for richer households compared to poorer households and a decrease in the predicted prevalence rate from the first to the most recent observation. However, the picture is very heterogeneous between countries, regarding levels, wealth gaps, and trends.

**Table 1:** Prevalence of diarrhoea across wealth quartiles in first and most recent survey year by country*

	Year	WQ 1	WQ 2	WQ 3	WQ 4	Diff	Ratio
Benin	1996	0.172 (0.167 to 0.177)	0.167 (0.161 to 0.173)	0.159 (0.153 to 0.166)	0.144 (0.136 to 0.152)	0.028	1.194
	2017	0.124 (0.123 to 0.125)	0.112 (0.111 to 0.114)	0.107 (0.106 to 0.108)	0.086 (0.084 to 0.087)	0.039	1.451
Burkina Faso	1998	0.199 (0.196 to 0.202)	0.210 (0.207 to 0.212)	0.203 (0.200 to 0.207)	0.201 (0.198 to 0.205)	-0.003	0.986
	2010	0.146 (0.144 to 0.148)	0.157 (0.155 to 0.158)	0.150 (0.148 to 0.152)	0.148 (0.145 to 0.150)	-0.002	0.988
Cameroon	1998	0.160 (0.151 to 0.169)	0.131 (0.120 to 0.141)	0.104 (0.097 to 0.112)	0.085 (0.079 to 0.091)	0.074	1.874
	2014	0.262 (0.256 to 0.269)	0.205 (0.198 to 0.212)	0.162 (0.156 to 0.168)	0.127 (0.122 to 0.131)	0.136	2.070
Central African Republic	2000	0.267 (0.264 to 0.270)	0.255 (0.252 to 0.258)	0.252 (0.247 to 0.257)	0.255 (0.252 to 0.258)	0.011	1.045
	2010	0.253 (0.250 to 0.256)	0.236 (0.232 to 0.239)	0.234 (0.231 to 0.237)	0.238 (0.234 to 0.242)	0.015	1.062
Chad	1996	0.224 (0.221 to 0.227)	0.221 (0.217 to 0.225)	0.215 (0.211 to 0.219)			
	2014	0.226 (0.223 to 0.229)	0.225 (0.222 to 0.228)	0.239 (0.236 to 0.242)	0.215 (0.212 to 0.218)	0.011	1.050
Congo	2005	0.145 (0.140 to 0.150)	0.148 (0.143 to 0.154)	0.139 (0.133 to 0.145)	0.136 (0.131 to 0.142)	0.009	1.064
	2014	0.175 (0.172 to 0.178)	0.186 (0.182 to 0.190)	0.180 (0.175 to 0.185)	0.168 (0.161 to 0.175)	0.007	1.041
Congo Democratic Republic	2001	0.204 (0.201 to 0.208)	0.227 (0.223 to 0.230)	0.229 (0.227 to 0.231)			
	2013	0.167 (0.165 to 0.169)	0.175 (0.172 to 0.177)	0.180 (0.177 to 0.182)	0.171 (0.168 to 0.174)	-0.004	0.976
Cote d'Ivoire	1998	0.235 (0.227 to 0.242)	0.241 (0.235 to 0.247)	0.220 (0.212 to 0.228)	0.205 (0.199 to 0.211)	0.030	1.144
	2016	0.166 (0.164 to 0.169)	0.171 (0.168 to 0.174)	0.150 (0.146 to 0.153)	0.128 (0.123 to 0.133)	0.038	1.301
Gabon	2000	0.183 (0.178 to 0.188)	0.179 (0.172 to 0.186)	0.185 (0.178 to 0.193)	0.147 (0.140 to 0.154)	0.036	1.248
	2012	0.193 (0.187 to 0.198)	0.179 (0.172 to 0.186)	0.177 (0.167 to 0.186)	0.135 (0.126 to 0.144)	0.058	1.429
Gambia	2000	0.214 (0.209 to 0.220)	0.220 (0.215 to 0.225)	0.215 (0.209 to 0.221)	0.205 (0.198 to 0.213)	0.009	1.045
	2013	0.181 (0.178 to 0.184)	0.185 (0.182 to 0.189)	0.177 (0.173 to 0.182)	0.164 (0.157 to 0.170)	0.017	1.106
Ghana	1998	0.211 (0.208 to 0.214)	0.180 (0.176 to 0.184)	0.181 (0.176 to 0.186)	0.132 (0.127 to 0.137)	0.078	1.594
	2014	0.155 (0.153 to 0.158)	0.120 (0.117 to 0.123)	0.117 (0.114 to 0.120)	0.065 (0.060 to 0.069)	0.091	2.398
Guinea	1999	0.219 (0.216 to 0.222)	0.217 (0.214 to 0.220)	0.216 (0.213 to 0.220)	0.222 (0.219 to 0.226)	-0.003	0.985
	2016	0.108 (0.105 to 0.110)	0.102 (0.099 to 0.105)	0.099 (0.096 to 0.102)	0.101 (0.098 to 0.105)	0.006	1.063
Guinea-Bissau	2006	0.123 (0.120 to 0.126)	0.122 (0.118 to 0.126)	0.129 (0.124 to 0.133)	0.139 (0.135 to 0.143)	-0.016	0.886
	2014	0.113 (0.111 to 0.115)	0.120 (0.118 to 0.123)	0.125 (0.122 to 0.128)	0.130 (0.126 to 0.135)	-0.017	0.867
Liberia	2006	0.208 (0.204 to 0.212)	0.223 (0.218 to 0.228)	0.221 (0.216 to 0.225)	0.191 (0.184 to 0.199)	0.017	1.088
	2013	0.234 (0.231 to 0.237)	0.239 (0.234 to 0.243)	0.235 (0.230 to 0.240)	0.215 (0.208 to 0.222)	0.019	1.091
Mali	1995	0.170 (0.165 to 0.175)	0.169 (0.163 to 0.174)	0.165 (0.160 to 0.169)	0.142 (0.138 to 0.146)	0.028	1.196
	2015	0.163 (0.161 to 0.165)	0.159 (0.158 to 0.161)	0.149 (0.148 to 0.151)	0.117 (0.115 to 0.119)	0.046	1.388
Mauritania	2007	0.234 (0.232 to 0.236)	0.225 (0.222 to 0.227)	0.213 (0.211 to 0.215)	0.185 (0.183 to 0.188)	0.049	1.265
	2015	0.209 (0.207 to 0.211)	0.198 (0.196 to 0.200)	0.188 (0.186 to 0.190)	0.161 (0.158 to 0.164)	0.048	1.299
Niger	1998	0.265 (0.259 to 0.272)	0.254 (0.248 to 0.261)	0.228 (0.221 to 0.234)			
	2012	0.163 (0.161 to 0.166)	0.142 (0.139 to 0.144)	0.144 (0.141 to 0.147)	0.122 (0.118 to 0.126)	0.041	1.335
Nigeria	1999	0.118 (0.114 to 0.123)	0.102 (0.097 to 0.106)	0.083 (0.080 to 0.086)	0.068 (0.064 to 0.071)	0.050	1.742
	2013	0.151 (0.149 to 0.152)	0.114 (0.112 to 0.117)	0.073 (0.071 to 0.075)	0.056 (0.054 to 0.058)	0.095	2.715
Sao Tome and Principe	2000	0.179 (0.171 to 0.186)	0.186 (0.177 to 0.196)	0.184 (0.176 to 0.191)	0.160 (0.150 to 0.170)	0.019	1.116
	2014	0.190 (0.183 to 0.197)	0.186 (0.180 to 0.191)	0.184 (0.176 to 0.191)	0.175 (0.165 to 0.184)	0.016	1.089
Senegal	1997	0.180 (0.177 to 0.183)	0.154 (0.151 to 0.158)	0.148 (0.144 to 0.152)	0.125 (0.120 to 0.129)	0.055	1.444
	2017	0.208 (0.206 to 0.210)	0.183 (0.180 to 0.186)	0.174 (0.170 to 0.177)	0.153 (0.148 to 0.157)	0.055	1.360
Sierra Leone	2000	0.267 (0.264 to 0.270)	0.269 (0.266 to 0.273)	0.253 (0.249 to 0.257)	0.249 (0.244 to 0.255)	0.018	1.072
	2017	0.087 (0.085 to 0.088)	0.085 (0.084 to 0.087)	0.071 (0.069 to 0.073)	0.066 (0.064 to 0.068)	0.021	1.310
Togo	1998	0.218 (0.211 to 0.224)	0.198 (0.191 to 0.204)	0.190 (0.180 to 0.201)	0.166 (0.159 to 0.173)	0.052	1.313
	2013	0.191 (0.188 to 0.194)	0.165 (0.161 to 0.169)	0.142 (0.138 to 0.147)	0.100 (0.096 to 0.104)	0.091	1.903

*Note: Predicted prevalence of diarrhoea across wealth quartiles for each country in the first and most recent survey. Predicted prevalence is calculated from a country-specific regression of disease on wealth controlling for child’s gender and age in years, mother’s age at birth of the child, mother’s educational level, location of the household (rural/urban), and survey-year fixed effects. 95% confidence intervals in parentheses. Column “Diff” contains the percentage point difference between the predicted prevalence in the poorest and richest wealth quartile. Column “Ratio” contains the ratio of the predicted prevalence rate in the poorest to that in the richest quartile (prevalence WQ1 / prevalence WQ4).

**Table 2:** Prevalence of ARI across wealth quartiles in first and most recent survey year by country*

	Year	WQ 1	WQ 2	WQ 3	WQ 4	Diff	Ratio
Benin	1996	0.101 (0.098 to 0.104)	0.100 (0.096 to 0.103)	0.096 (0.092 to 0.100)	0.089 (0.084 to 0.094)	0.012	1.133
	2017	0.065 (0.064 to 0.066)	0.059 (0.058 to 0.059)	0.057 (0.056 to 0.057)	0.050 (0.049 to 0.050)	0.015	1.309
Burkina Faso	1998	0.140 (0.139 to 0.141)	0.146 (0.144 to 0.147)	0.136 (0.135 to 0.138)	0.123 (0.121 to 0.124)	0.017	1.142
	2010	0.044 (0.044 to 0.045)	0.051 (0.050 to 0.051)	0.041 (0.041 to 0.042)	0.029 (0.028 to 0.030)	0.016	1.535
Cameroon	1998	0.131 (0.125 to 0.136)	0.131 (0.124 to 0.138)	0.120 (0.114 to 0.125)	0.126 (0.120 to 0.131)	0.005	1.039
	2014	0.179 (0.178 to 0.181)	0.174 (0.172 to 0.176)	0.155 (0.154 to 0.157)	0.167 (0.165 to 0.169)	0.013	1.076
Central African Republic	2000	0.269 (0.258 to 0.280)	0.263 (0.251 to 0.275)	0.256 (0.243 to 0.269)	0.234 (0.223 to 0.244)	0.036	1.152
	2010	0.314 (0.313 to 0.316)	0.295 (0.293 to 0.297)	0.282 (0.280 to 0.284)	0.277 (0.274 to 0.279)	0.038	1.136
Chad	1996	0.130 (0.129 to 0.131)	0.129 (0.127 to 0.130)	0.130 (0.128 to 0.132)			
	2014	0.116 (0.115 to 0.117)	0.114 (0.113 to 0.115)	0.119 (0.117 to 0.120)	0.114 (0.113 to 0.115)	0.002	1.014
Congo	2005	0.099 (0.096 to 0.102)	0.078 (0.076 to 0.080)	0.073 (0.070 to 0.075)	0.074 (0.072 to 0.076)	0.025	1.332
	2014	0.155 (0.153 to 0.156)	0.133 (0.132 to 0.135)	0.129 (0.128 to 0.131)	0.129 (0.127 to 0.131)	0.026	1.201
Congo Democratic Republic	2001	0.190 (0.173 to 0.206)	0.227 (0.211 to 0.244)	0.222 (0.209 to 0.236)			
	2013	0.136 (0.135 to 0.137)	0.147 (0.146 to 0.149)	0.156 (0.153 to 0.158)	0.106 (0.104 to 0.108)	0.030	1.283
Cote d'Ivoire	1998	0.177 (0.173 to 0.181)	0.187 (0.183 to 0.190)	0.166 (0.161 to 0.171)	0.160 (0.156 to 0.164)	0.017	1.108
	2016	0.081 (0.080 to 0.083)	0.091 (0.090 to 0.093)	0.071 (0.069 to 0.072)	0.067 (0.065 to 0.069)	0.015	1.220
Gabon	2000	0.136 (0.134 to 0.138)	0.126 (0.122 to 0.130)	0.162 (0.159 to 0.166)	0.147 (0.142 to 0.151)	-0.011	0.928
	2012	0.165 (0.161 to 0.168)	0.153 (0.150 to 0.156)	0.182 (0.176 to 0.188)	0.170 (0.165 to 0.174)	-0.005	0.970
Gambia	2000	0.115 (0.102 to 0.128)	0.114 (0.102 to 0.127)	0.106 (0.093 to 0.119)	0.104 (0.091 to 0.118)	0.011	1.105
	2013	0.087 (0.085 to 0.088)	0.094 (0.092 to 0.096)	0.079 (0.077 to 0.081)	0.093 (0.090 to 0.095)	-0.006	0.934
Ghana	1998	0.147 (0.146 to 0.148)	0.145 (0.143 to 0.146)	0.142 (0.140 to 0.144)	0.122 (0.120 to 0.124)	0.025	1.205
	2014	0.079 (0.078 to 0.080)	0.074 (0.073 to 0.075)	0.072 (0.069 to 0.074)	0.050 (0.048 to 0.051)	0.029	1.594
Guinea	1999	0.174 (0.172 to 0.175)	0.166 (0.165 to 0.168)	0.163 (0.162 to 0.165)	0.151 (0.150 to 0.153)	0.022	1.148
	2016	0.088 (0.087 to 0.089)	0.080 (0.078 to 0.081)	0.077 (0.076 to 0.078)	0.064 (0.062 to 0.066)	0.024	1.373
Guinea-Bissau	2006	0.075 (0.074 to 0.076)	0.055 (0.053 to 0.057)	0.072 (0.069 to 0.075)	0.094 (0.091 to 0.097)	-0.019	0.794
	2014	0.057 (0.056 to 0.058)	0.043 (0.041 to 0.045)	0.064 (0.062 to 0.067)	0.082 (0.079 to 0.085)	-0.025	0.696
Liberia	2006	0.142 (0.140 to 0.144)	0.130 (0.127 to 0.134)	0.126 (0.122 to 0.130)	0.119 (0.114 to 0.124)	0.023	1.191
	2013	0.131 (0.129 to 0.134)	0.111 (0.107 to 0.114)	0.104 (0.100 to 0.108)	0.109 (0.106 to 0.112)	0.022	1.203
Mali	1995	0.096 (0.093 to 0.099)	0.096 (0.093 to 0.099)	0.099 (0.096 to 0.102)	0.098 (0.095 to 0.101)	-0.002	0.978
	2015	0.055 (0.055 to 0.055)	0.055 (0.054 to 0.055)	0.058 (0.057 to 0.058)	0.057 (0.057 to 0.058)	-0.002	0.962
Mauritania	2007	0.087 (0.087 to 0.088)	0.090 (0.089 to 0.091)	0.099 (0.098 to 0.100)	0.108 (0.107 to 0.110)	-0.021	0.806
	2015	0.063 (0.062 to 0.063)	0.064 (0.063 to 0.065)	0.074 (0.073 to 0.075)	0.083 (0.081 to 0.084)	-0.020	0.756
Niger	1998	0.090 (0.088 to 0.093)	0.108 (0.105 to 0.111)	0.092 (0.089 to 0.094)			
	2012	0.073 (0.072 to 0.074)	0.071 (0.070 to 0.072)	0.097 (0.096 to 0.098)	0.079 (0.078 to 0.080)	-0.006	0.923
Nigeria	1999	0.073 (0.071 to 0.076)	0.074 (0.071 to 0.077)	0.063 (0.061 to 0.066)	0.056 (0.053 to 0.059)	0.017	1.312
	2013	0.053 (0.052 to 0.053)	0.049 (0.049 to 0.050)	0.026 (0.025 to 0.026)	0.018 (0.017 to 0.018)	0.035	2.957
Sao Tome and Principe	2000	0.163 (0.143 to 0.183)	0.182 (0.159 to 0.206)	0.155 (0.135 to 0.176)	0.143 (0.118 to 0.168)	0.020	1.140
	2014	0.253 (0.250 to 0.257)	0.259 (0.255 to 0.263)	0.251 (0.246 to 0.255)	0.236 (0.232 to 0.240)	0.017	1.074
Senegal	2000	0.182 (0.174 to 0.191)	0.174 (0.164 to 0.185)	0.158 (0.145 to 0.171)	0.154 (0.133 to 0.175)	0.028	1.185
	2017	0.068 (0.067 to 0.068)	0.070 (0.069 to 0.071)	0.090 (0.088 to 0.092)	0.085 (0.083 to 0.087)	-0.017	0.795
Sierra Leone	2000	0.199 (0.184 to 0.215)	0.204 (0.190 to 0.218)	0.181 (0.163 to 0.198)	0.177 (0.160 to 0.194)	0.022	1.125
	2017	0.060 (0.059 to 0.060)	0.064 (0.063 to 0.065)	0.058 (0.057 to 0.059)	0.035 (0.034 to 0.037)	0.024	1.685
Togo	1998	0.137 (0.133 to 0.140)	0.121 (0.117 to 0.124)	0.125 (0.119 to 0.132)	0.122 (0.117 to 0.127)	0.015	1.122
	2013	0.160 (0.159 to 0.161)	0.139 (0.137 to 0.140)	0.141 (0.139 to 0.143)	0.135 (0.134 to 0.137)	0.025	1.184

*Note: Predicted prevalence of ARI across wealth quartiles for each country in the first and most recent survey. Predicted prevalence is calculated from a country-specific regression of disease on wealth controlling for child’s gender and age in years, mother’s age at birth of the child, mother’s educational level, location of the household (rural/urban), and survey-year fixed effects. 95% confidence intervals in parentheses. Column “Diff” contains the percentage point difference between the predicted prevalence in the poorest and richest wealth quartile. Column “Ratio” contains the ratio of the predicted prevalence rate in the poorest to that in the richest quartile (prevalence WQ1 / prevalence WQ4). ARI – acute respiratory infections.

**Table 3:** Prevalence of fever across wealth quartiles in first and most recent survey year by country*

	Year	WQ 1	WQ 2	WQ 3	WQ 4	Diff	Ratio
Benin	1996	0.341 (0.331 to 0.352)	0.352 (0.339 to 0.364)	0.335 (0.321 to 0.349)	0.301 (0.284 to 0.318)	0.040	1.133
	2017	0.210 (0.209 to 0.212)	0.218 (0.216 to 0.220)	0.205 (0.203 to 0.207)	0.158 (0.156 to 0.160)	0.052	1.328
Burkina Faso	1998	0.370 (0.367 to 0.373)	0.368 (0.365 to 0.371)	0.370 (0.367 to 0.374)	0.359 (0.355 to 0.363)	0.011	1.031
	2010	0.212 (0.210 to 0.214)	0.210 (0.208 to 0.212)	0.213 (0.211 to 0.214)	0.203 (0.200 to 0.205)	0.009	1.046
Cameroon	1998	0.190 (0.182 to 0.199)	0.202 (0.191 to 0.213)	0.197 (0.188 to 0.205)	0.185 (0.177 to 0.193)	0.005	1.028
	2014	0.256 (0.254 to 0.259)	0.269 (0.267 to 0.272)	0.249 (0.246 to 0.252)	0.239 (0.237 to 0.242)	0.017	1.071
Central African Republic	2000	0.331 (0.329 to 0.334)	0.311 (0.309 to 0.314)	0.311 (0.306 to 0.316)	0.316 (0.314 to 0.319)	0.015	1.048
	2010	0.335 (0.333 to 0.337)	0.312 (0.309 to 0.314)	0.314 (0.311 to 0.316)	0.323 (0.320 to 0.325)	0.012	1.038
Chad	1996	0.327 (0.324 to 0.331)	0.311 (0.307 to 0.316)	0.343 (0.338 to 0.348)			
	2014	0.238 (0.235 to 0.242)	0.249 (0.246 to 0.252)	0.238 (0.234 to 0.241)	0.249 (0.246 to 0.252)	-0.011	0.956
Congo	2005	0.264 (0.261 to 0.267)	0.233 (0.230 to 0.236)	0.245 (0.242 to 0.248)	0.226 (0.222 to 0.229)	0.039	1.172
	2014	0.321 (0.319 to 0.323)	0.289 (0.287 to 0.291)	0.304 (0.301 to 0.306)	0.285 (0.281 to 0.288)	0.036	1.127
Congo Democratic Republic	2001	0.389 (0.384 to 0.394)	0.418 (0.415 to 0.421)	0.419 (0.418 to 0.421)			
	2013	0.307 (0.305 to 0.309)	0.318 (0.316 to 0.320)	0.317 (0.315 to 0.319)	0.271 (0.269 to 0.274)	0.036	1.131
Cote d'Ivoire	1998	0.389 (0.382 to 0.396)	0.404 (0.398 to 0.411)	0.363 (0.352 to 0.374)	0.358 (0.350 to 0.365)	0.031	1.088
	2016	0.278 (0.276 to 0.280)	0.294 (0.291 to 0.296)	0.256 (0.253 to 0.258)	0.243 (0.239 to 0.247)	0.035	1.142
Gabon	2000	0.313 (0.308 to 0.317)	0.307 (0.301 to 0.312)	0.334 (0.328 to 0.340)	0.316 (0.311 to 0.322)	-0.004	0.988
	2012	0.254 (0.249 to 0.259)	0.244 (0.241 to 0.248)	0.260 (0.252 to 0.268)	0.249 (0.243 to 0.256)	0.004	1.017
Gambia	2000	0.143 (0.141 to 0.145)	0.149 (0.147 to 0.150)	0.139 (0.137 to 0.141)	0.148 (0.145 to 0.150)	-0.005	0.969
	2013	0.119 (0.118 to 0.120)	0.123 (0.122 to 0.125)	0.117 (0.116 to 0.118)	0.124 (0.122 to 0.126)	-0.005	0.958
Ghana	1998	0.293 (0.291 to 0.296)	0.281 (0.278 to 0.285)	0.272 (0.268 to 0.277)	0.236 (0.232 to 0.240)	0.058	1.245
	2014	0.165 (0.163 to 0.167)	0.145 (0.142 to 0.148)	0.139 (0.135 to 0.142)	0.100 (0.096 to 0.104)	0.065	1.654
Guinea	1999	0.452 (0.448 to 0.457)	0.445 (0.440 to 0.449)	0.430 (0.425 to 0.436)	0.393 (0.388 to 0.399)	0.059	1.150
	2016	0.216 (0.213 to 0.219)	0.203 (0.200 to 0.206)	0.190 (0.186 to 0.193)	0.150 (0.145 to 0.154)	0.067	1.446
Guinea-Bissau	2006	0.128 (0.126 to 0.130)	0.116 (0.113 to 0.119)	0.140 (0.135 to 0.145)	0.154 (0.150 to 0.158)	-0.026	0.829
	2014	0.149 (0.147 to 0.151)	0.145 (0.142 to 0.147)	0.171 (0.167 to 0.174)	0.179 (0.176 to 0.182)	-0.030	0.834
Liberia	2006	0.318 (0.313 to 0.323)	0.331 (0.326 to 0.337)	0.355 (0.351 to 0.358)	0.325 (0.319 to 0.330)	-0.007	0.980
	2013	0.289 (0.287 to 0.292)	0.297 (0.294 to 0.300)	0.317 (0.312 to 0.322)	0.289 (0.283 to 0.295)	-0.000	1.000
Mali	1995	0.251 (0.244 to 0.258)	0.251 (0.243 to 0.259)	0.253 (0.245 to 0.260)	0.233 (0.226 to 0.239)	0.019	1.080
	2015	0.161 (0.160 to 0.162)	0.159 (0.158 to 0.161)	0.156 (0.155 to 0.157)	0.127 (0.126 to 0.129)	0.034	1.265
Mauritania	2007	0.181 (0.179 to 0.182)	0.170 (0.168 to 0.171)	0.160 (0.158 to 0.161)	0.161 (0.159 to 0.162)	0.020	1.125
	2015	0.207 (0.206 to 0.209)	0.197 (0.195 to 0.198)	0.184 (0.183 to 0.186)	0.183 (0.181 to 0.186)	0.024	1.131
Niger	1998	0.339 (0.330 to 0.347)	0.331 (0.323 to 0.340)	0.285 (0.277 to 0.294)			
	2012	0.167 (0.166 to 0.169)	0.153 (0.151 to 0.154)	0.154 (0.151 to 0.156)	0.108 (0.104 to 0.111)	0.060	1.556
Nigeria	1999	0.194 (0.187 to 0.201)	0.189 (0.181 to 0.198)	0.189 (0.183 to 0.196)	0.157 (0.149 to 0.165)	0.037	1.233
	2013	0.155 (0.154 to 0.157)	0.138 (0.136 to 0.139)	0.119 (0.118 to 0.121)	0.078 (0.077 to 0.080)	0.077	1.981
Sao Tome and Principe	2000	0.266 (0.258 to 0.274)	0.276 (0.267 to 0.285)	0.258 (0.250 to 0.265)	0.261 (0.251 to 0.272)	0.004	1.017
	2014	0.274 (0.266 to 0.281)	0.269 (0.263 to 0.275)	0.258 (0.250 to 0.265)	0.279 (0.270 to 0.287)	-0.005	0.982
Senegal	2000	0.207 (0.201 to 0.212)	0.197 (0.191 to 0.202)	0.212 (0.206 to 0.217)	0.201 (0.195 to 0.206)	0.006	1.030
	2017	0.213 (0.212 to 0.214)	0.194 (0.192 to 0.196)	0.209 (0.206 to 0.212)	0.198 (0.195 to 0.201)	0.015	1.077
Sierra Leone	2000	0.468 (0.463 to 0.472)	0.489 (0.486 to 0.493)	0.478 (0.474 to 0.481)	0.474 (0.465 to 0.482)	-0.006	0.987
	2017	0.202 (0.200 to 0.203)	0.219 (0.217 to 0.220)	0.210 (0.208 to 0.211)	0.204 (0.202 to 0.206)	-0.003	0.987
Togo	1998	0.260 (0.253 to 0.267)	0.241 (0.233 to 0.249)	0.223 (0.211 to 0.236)	0.196 (0.187 to 0.204)	0.064	1.329
	2013	0.265 (0.263 to 0.267)	0.241 (0.238 to 0.243)	0.203 (0.200 to 0.206)	0.155 (0.153 to 0.158)	0.110	1.705

*Note: Predicted prevalence of fever across wealth quartiles for each country in the first and most recent survey. Predicted prevalence is calculated from a country-specific regression of disease on wealth controlling for child’s gender and age in years, mother’s age at birth of the child, mother’s educational level, location of the household (rural/urban), and survey-year fixed effects. 95% confidence intervals in parentheses. Column “Diff” contains the percentage point difference between the predicted prevalence in the poorest and richest wealth quartile. Column “Ratio” contains the ratio of the predicted prevalence rate in the poorest to that in the richest quartile (prevalence WQ1 / prevalence WQ4).

**Table 4:** Prevalence of malaria across wealth quartiles in most recent survey year by country*

	Year	WQ 1	WQ 2	WQ 3	WQ 4	Diff	Ratio
Benin	2017	0.206 (0.193 to 0.218)	0.187 (0.177 to 0.197)	0.154 (0.145 to 0.164)	0.074 (0.069 to 0.080)	0.131	2.763
Burkina Faso	2010	0.355 (0.338 to 0.372)	0.359 (0.341 to 0.377)	0.327 (0.309 to 0.345)	0.243 (0.227 to 0.260)	0.111	1.457
Congo Democratic Republic	2013	0.165 (0.159 to 0.171)	0.146 (0.137 to 0.155)	0.123 (0.114 to 0.132)	0.076 (0.069 to 0.082)	0.089	2.183
Gambia	2013	0.009 (0.008 to 0.010)	0.012 (0.010 to 0.014)	0.006 (0.005 to 0.008)	0.005 (0.004 to 0.006)	0.004	1.796
Ghana	2014	0.269 (0.252 to 0.286)	0.177 (0.157 to 0.196)	0.108 (0.094 to 0.122)	0.008 (0.001 to 0.015)	0.261	34.136
Senegal	2017	0.024 (0.024 to 0.025)	0.005 (0.004 to 0.005)	-0.001 (-0.001 to 0.000)	-0.002 (-0.003 to 0.002)	0.026	-11.710
Togo	2013	0.249 (0.234 to 0.263)	0.205 (0.189 to 0.222)	0.127 (0.114 to 0.140)	0.047 (0.038 to 0.055)	0.202	5.339

*Note: Predicted prevalence of malaria across wealth quartiles for each country in the most recent survey. Predicted prevalence is calculated from a country-specific regression of disease on wealth controlling for child’s gender and age in years, mother’s age at birth of the child, mother’s educational level, location of the household (rural/urban), and survey-year fixed effects. 95% confidence intervals in parentheses. Column “Diff” contains the percentage point difference between the predicted prevalence in the poorest and richest wealth quartile. Column “Ratio” contains the ratio of the predicted prevalence rate in the poorest to that in the richest quartile (prevalence WQ1 / prevalence WQ4).

For the most recent survey, the predicted prevalence rate of diarrhoea among children from the poorest quartile ranges from 8.7% in Sierra Leone to 26.2% in Cameroon. For children from the richest quartile it ranges from 5.6% in Nigeria to 23.8% in the Central African Republic. From the first to the most recent survey, the prevalence of diarrhoea increased by about 10 percentage points in the poorest wealth quartile in Cameroon and declined by about 18 percentage points for all quartiles in Sierra Leone.

The predicted prevalence rate of ARI in the most recent period ranges from 4.4% in Burkina Faso to 31.4% in the Central African Republic for the first quartile and from 1.8% in Nigeria to 27.7% also in the Central African Republic for children from the fourth quartile. The prevalence increased over time by 9.6 percentage points in the third quartile in Sao Tome and Principe and decreased by 14.2 percentage points for children of the richest quartile in Sierra Leone.

The highest prevalence of fever is observed in the Central African Republic with 33.5% for the poorest and 32.3% for the richest quartile, the lowest in Gambia with 11.9% and Nigeria with 7.8% for children from the poorest and the richest quartile respectively. The change in the prevalence over time ranges from a just above 5 percentage point increase in the richest quartile in Cameroon to around 27 percentage point decrease for all quartiles in Sierra Leone.

The broadest range is observed for the prevalence of malaria from 0.9% in Gambia to 35.5% in Burkina Faso for children from the first quartile and 0 to 24.3% for children from the fourth quartile in the same countries.

We express the wealth gradient with two different indicators shown in **Tables 1** to **4**. Column 6 contains the wealth gradient in terms of the percentage point difference in prevalence between the poorest and the richest quartile. Column 7 shows the ratio of the prevalence rate in the poorest to that in the richest quartile, accounting for the level of the prevalence.

For diarrhoea, the gradient in the recent period is largest in Nigeria with a percentage point difference of 9.5 and a ratio of 2.7. Guinea-Bissau, Burkina Faso and the Democratic Republic of Congo show a negative gradient. Nigeria also has the biggest ratio for the prevalence of ARI, with children from poorer households being almost 3 times more likely to suffer from acute respiratory disease than children from the richest quartile. The Central African Republic has the largest percentage point difference with 3.8 percentage points. Similarly, Nigeria has the largest gradient for the prevalence of fever with a ratio of almost 2. In Togo, the percentage point difference is largest with children from the poorest quartile having a 11 percentage point higher likelihood of suffering from fever compared to children from the richest wealth quartile. Ghana has the biggest gradient for malaria with a difference of 26 percentage points and a ratio of 34, an extreme case of a wealth gradient.

**Figure 3:**
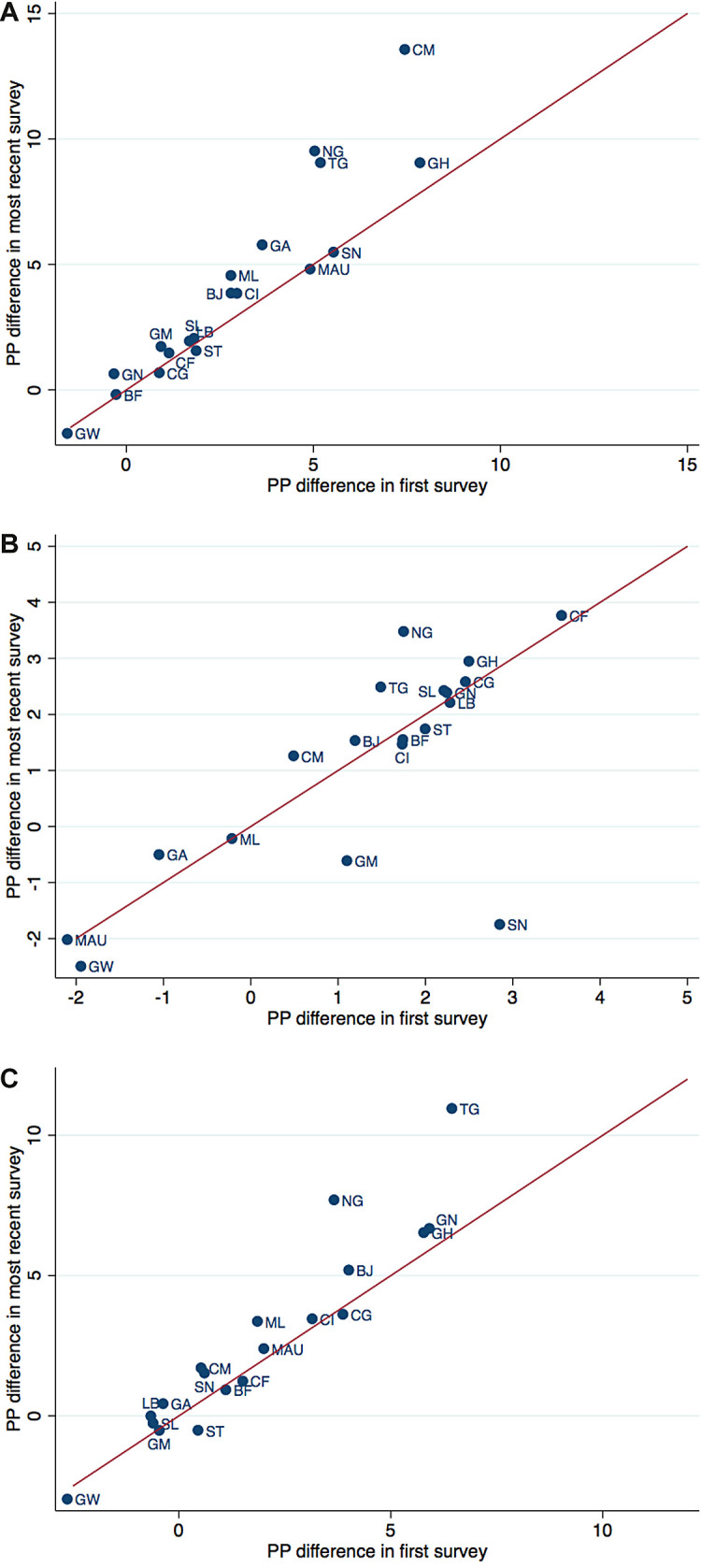
Change in wealth gap in disease prevalence, expressed as percentage point difference. **Panel A.** Change in wealth gap in diarrhoea prevalence, expressed as percentage point difference. Note: Percentage point difference between predicted prevalence of diarrhoea in poorest and richest wealth quartile, in first survey and most recent survey of each country. Reflects country specific wealth gap given in column “Diff” of **Table 1****. Panel B.** Change in wealth gap in ARI prevalence, expressed as percentage point difference. Note: Percentage point difference between predicted prevalence of ARI in poorest and richest wealth quartile, in first survey and most recent survey of each country. Reflects country specific wealth gap given in column “Diff” of **Table 2**. ARI – acute respiratory infections. **Panel C.** Change in wealth gap in fever prevalence, expressed as percentage point difference. Note: Percentage point difference between predicted prevalence of fever in poorest and richest wealth quartile, in first survey and most recent survey of each country. Reflects country specific wealth gap given in column “Diff” of **Table 3****.**

**Figure 4:**
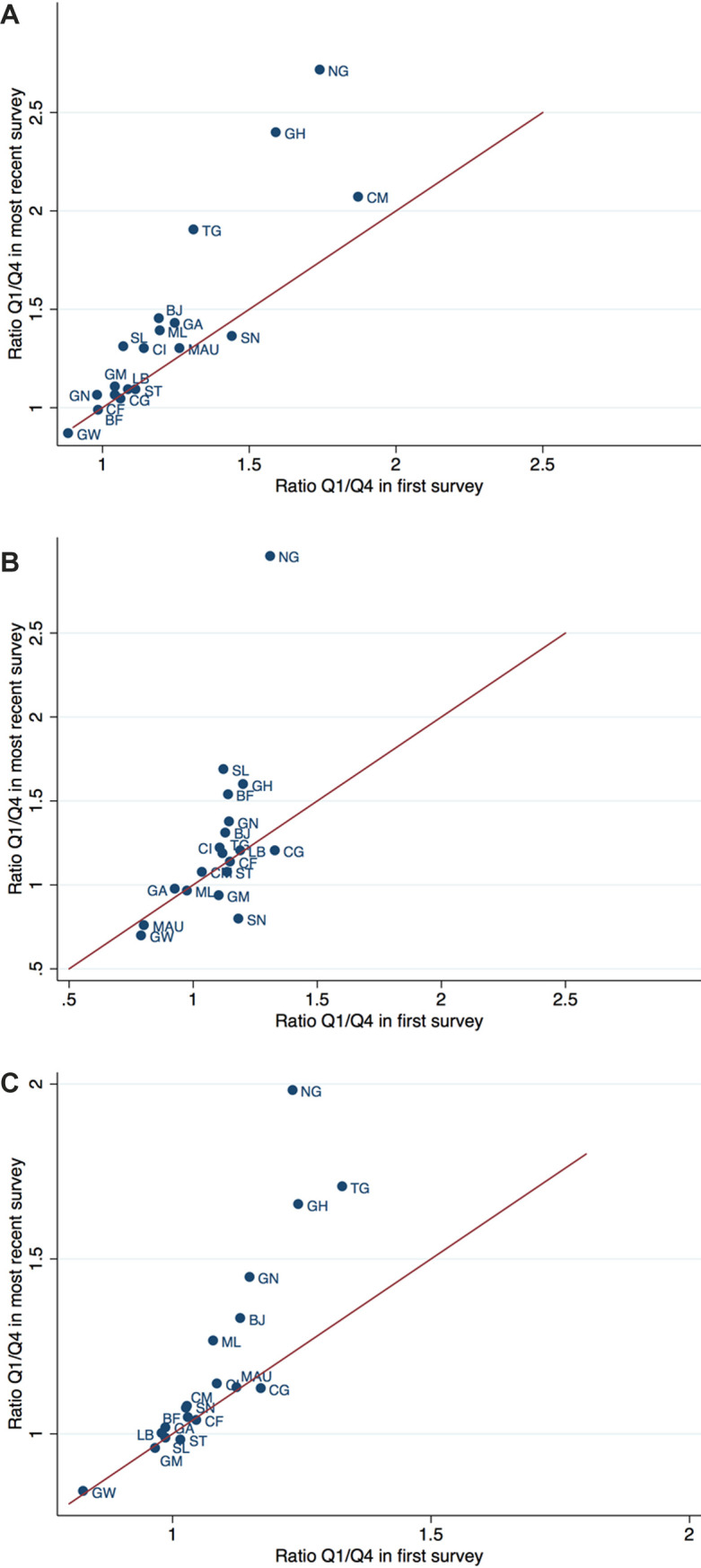
Change in wealth gap in disease prevalence, expressed as ratio Q1/Q4. **Panel A.** Change in wealth gap in diarrhoea prevalence, expressed as ratio Q1/Q4. Note: Ratio of predicted prevalence of diarrhoea in poorest to that in the richest wealth quartile, in first survey and most recent survey of each country. Reflects country specific wealth gap given in column “Ratio” of **Table 1****. Panel B.** Change in wealth gap in ARI prevalence, expressed as ratio Q1/Q4. Note: Ratio of predicted prevalence of ARI in poorest to that in the richest wealth quartile, in first survey and most recent survey of each country. Reflects country specific wealth gap given in column “Ratio” of **Table 2**. ARI – acute respiratory infections. **Panel C.** Change in wealth gap in fever prevalence, expressed as ratio Q1/Q4. Note: Ratio of predicted prevalence of fever in poorest to that in the richest wealth quartile, in first survey and most recent survey of each country. Reflects country specific wealth gap given in column “Ratio” of **Table 3****.**

The percentage point differences between the predicted prevalence of the poorest and the richest quartile in different countries and their change from the first to the last survey are illustrated in **Figure 3****,** Panels A-C. The difference between the poorest and the richest quartile in the earliest survey is plotted on the horizontal axis and the difference in the most recent survey on the vertical axis. For countries located above the 45-degree line, the difference between the riches and the poorest quartile increased over time, for those below it decreased. The distance to the line indicates the magnitude of the change. **Figure 4**, Panels A-C are similar to **Figure 3**, Panels A-C, but use the ratio of the prevalence in the poorest to those in the richest quartile instead of the difference, thereby accounting for the level of the prevalence. Country abbreviations are listed in **Table 5**.

**Table 5:** List of countries

Country name	Country code
Burkina Faso	BF
Benin	BJ
Congo Democratic Republic	CD
Central African Republic	CF
Congo	CG
Cote d'Ivoire	CI
Cameroon	CM
Equatorial Guinea	EQG
Gabon	GA
Ghana	GH
Gambia	GM
Guinea	GN
Guinea-Bissau	GW
Liberia	LB
Mauritania	MAU
Mali	ML
Nigeria	NG
Niger	NI
Sierra Leone	SL
Senegal	SN
Sao Tome and Principe	ST
Chad	TD
Togo	TG

In most countries, the wealth gradient of diarrhoea prevalence increased over time. The largest increase in terms of difference in percentage points occurred in Cameroon, Nigeria and Togo, and in terms of the ratio in Nigeria, Ghana and Togo. While some countries show a decline in the gradient of ARI, for the majority of countries it has grown over the years. The largest increase in the difference occurred in Nigeria, the largest decrease in Senegal and Gambia. The wealth gradient for the prevalence of fever amplified for most countries over time. Nigeria and Togo show a very large rise both in the difference as well as in the ratio. For Ghana with low overall prevalence of fever, the difference has not increased largely but the ratio did. All countries with a decreasing gap show only smaller changes.

## DISCUSSION

In this study, we analysed the change over time in the association between wealth status and childhood diseases for Western and Central African countries between 1995 and 2017. The predicted prevalence of each disease can be compared with the mean prevalence for the region [10].

Our results suggest a notable wealth gradient in the prevalence rates of diarrhoea, ARI, malaria, and fever. Children in the poorest 25 percent of households are much more often sick with diarrhoea, ARI, or malaria than children in the richest 25 percent. This is consistent with the findings of Kahabuka et al. in Tanzania [15]. While important reductions in prevalence rates have been achieved in the region, progress is slowing down. Moreover, these reductions have been unequally distributed. Overall, children in richer households have seen larger reductions in disease prevalence than children in poorer households, leading to an increasing wealth gradient. Despite substantial variation, this holds true for almost all countries included in our analysis. Looking at the prevalence of diarrhoea, 14 out of 22 countries have experienced a widening of the wealth gap measured both by the percentage point difference and the ratio between the poorest and wealthiest quartiles. A reduction in both indicators was found only for Congo and Sao Tome and Principe. The picture is only slightly better for the wealth gap in the prevalence of ARI. This finding might indicate that household wealth is becoming a more important risk factor for childhood diseases especially in the poorest households.

Programmes that have driven down disease prevalence are insufficiently capable of reaching the poorest, most vulnerable households. This may be because those households are the ones most difficult to reach or the ones with multiple interlinked challenges that were not yet addressed in their entirety by previous programmes. Efforts should be increased to adapt programmes to target the poorest households. Insufficient resources and inappropriate allocation are major impediments to the delivery of effective health care that reaches the poor. Most obviously, economic resources are often insufficient to support the provision of essential services. Public expenditures on health care have to be further scaled up substantially to address this concern.

A strong focus on financing primary health care as well as preventive measures seems warranted. Diarrhoea, pneumonia as well as malaria are all easily preventable diseases. For example, effective basic sanitation and drinking water infrastructure together with initiatives promoting healthy behaviour in the communities can substantially reduce the prevalence of diarrhoea. One of the key programmes of UNICEF West and Central Africa focuses on eliminating open defecation through community behaviour change and adequate sanitation systems [22].

This study contributes to the literature by providing insights about a particularly interesting region, containing some of the countries with the highest burden of the diarrhoea, ARI, and malaria. While predicting disease prevalence for the whole region, this study adds comparable analyses for each country separately. In addition, it expands the literature with the analysis of the change in the wealth gap over time.

There are some important limitations to this study. While we create an asset index that is comparable across countries and time to capture the wealth status of households, this asset index might not be the best suited for each individual country. Ensuring comparability at the same time means reducing the adaptation to each country context. In addition, as fewer assets were recorded in earlier surveys, the asset index is less accurate for the earlier time period than for the more recent periods. Data availability therefore limits the robustness of our analysis.

Moreover, surveys that did not record information on outcomes, assets and covariates had to be dropped. This restricts the generalisability of the results to the countries and years included in this analysis. Additionally, within included surveys, observations with missing information could not be used in the estimations, potentially limiting the representativeness of the sample.

Another limitation is that our analysis does not include all possible risk factors that might be associated with the prevalence of each disease. Our set of covariates in the prediction of disease prevalence is the same for each disease. This increases comparability between the estimations. However, we might miss out on disease specific risk factors such as nutrition for diarrhoea or housing characteristics for malaria. Our focus is on wealth and its association with childhood diseases. Analysing the effect size of any particular factor in isolation from the role of others, however, might lead to an overestimation of the true effect size [23].

## CONCLUSION

This study illustrates the strong association of wealth with the prevalence of childhood diseases in Western and Central African countries. It also highlights that the wealth gradient in health tends to increase rather than decrease over time, which raises concerns of increasing inequality that goes beyond wealth.

While increasing people’s wealth may impact several factors that in turn affect the prevalence of childhood diseases, this strategy is rather difficult and costly. Trying to reduce the link between wealth and childhood diseases, as well as directly aiming at reducing the prevalence of childhood diseases may be a more effective approach. In addition, households in the lowest wealth quartile might be burdened by several interlinked challenges at the same time that cannot be addressed by programmes focusing solely on disease prevention. This requires further efforts in implementing programmes that aim at improving health practices at the household and community level as well as programmes to improve housing, sanitation, and access to health services. The considerable wealth gradient suggests that targeting the poorest households and developing programmes that are adapted to their specific needs is necessary for achieving the goal of further reducing the high prevalence of childhood diseases and diminishing the stark health disparities across wealth.

## Additional material


Online Supplementary Document
