# Supplementary material for: The wealth gradient in diarrhoea, acute respiratory infections, and malaria in childhood over time: A descriptive analysis using DHS and MICS from Western and Central Africa between 1995 and 2017
Source: J Glob Health. 2021 Aug 10;11:13009. doi: 10.7189/jogh.11.13009 (PMC8397329; doi:10.7189/jogh.11.13009)
Supplement: Online Supplementary Document [file jogh-11-13009-s001.pdf]

Figure S1. Predicted prevalence of childhood diseases across wealth quartiles over time, disaggregated by gender.

Panel A. Predicted prevalence of diarrhoea across wealth quartiles over time, disaggregated by gender.

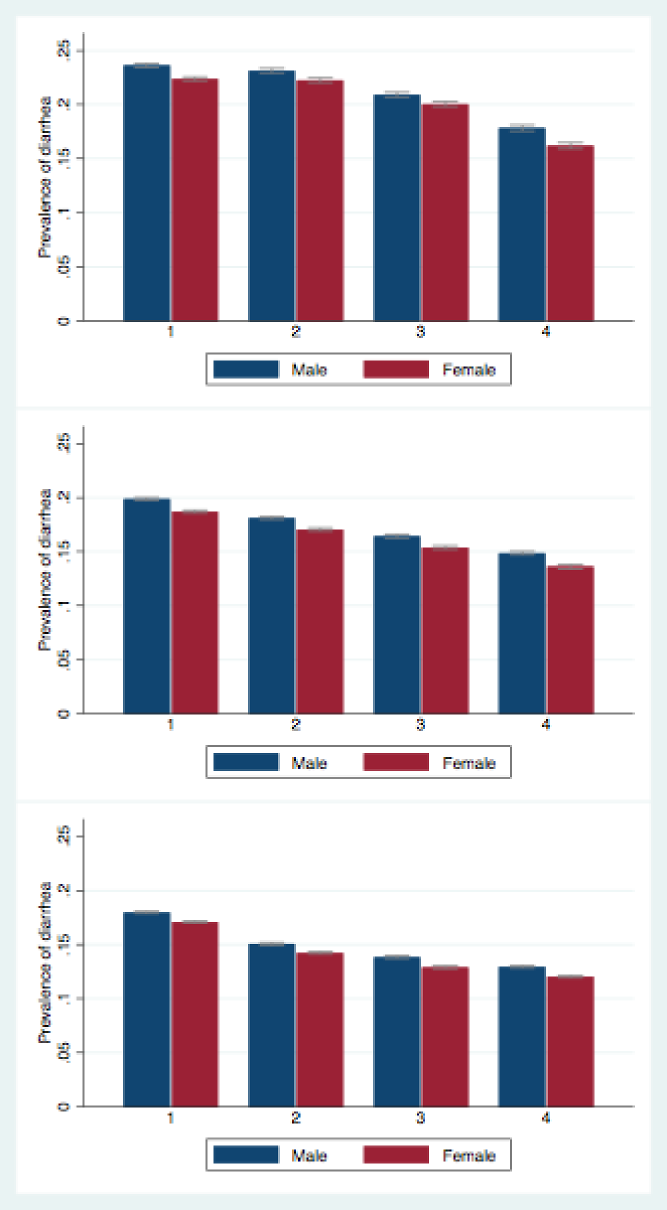

Note: Predicted prevalence of diarrhoea and 95% confidence intervals across wealth quartiles for all countries among boys and girls in three time periods: 1995-2001, 2002-2009, 2010-2017. Predicted prevalence is calculated separately for each time period from a regression of disease on wealth controlling for child's gender and age in years, mother's age at birth of the child, mother's educational level, location of the household (rural/urban), and survey-year and country-fixed effects. Observation weights are rescaled using each country's female population aged 15 to 49 years in the last year of the considered time period.

Panel B. Predicted prevalence of ARI across wealth quartiles over time, disaggregated by gender.

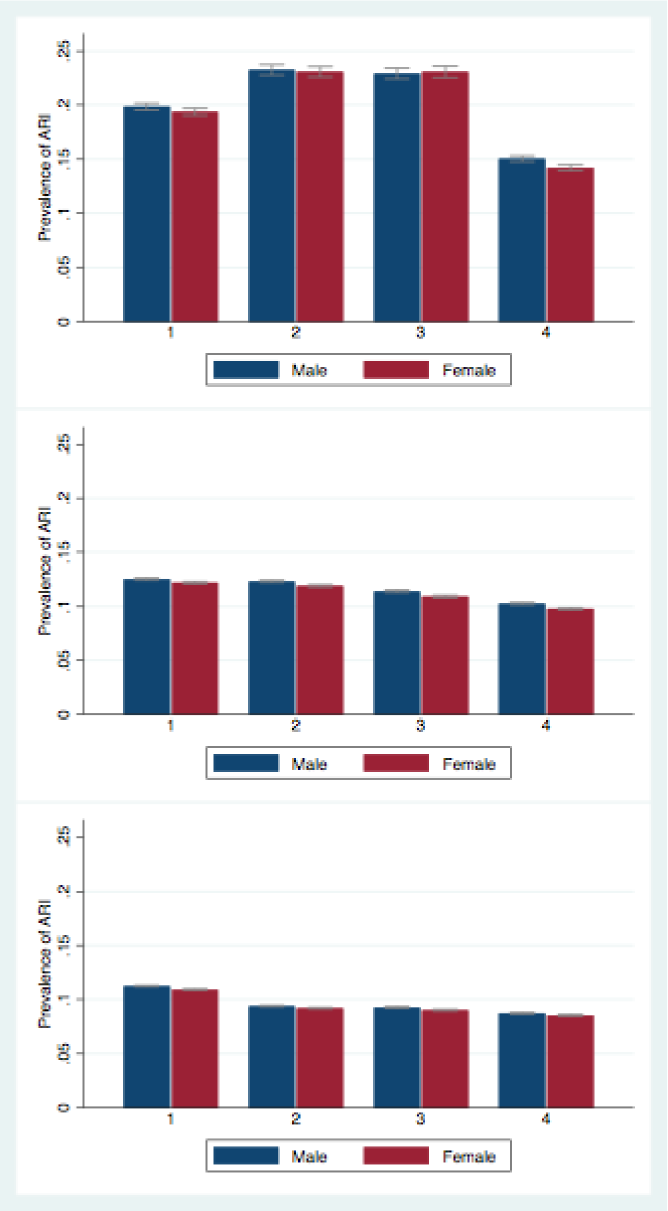

Note: Predicted prevalence of ARI and 95% confidence intervals across wealth quartiles for all countries among boys and girls in three time periods: 1995-2001, 2002-2009, 2010-2017. Predicted prevalence is calculated separately for each time period from a regression of disease on wealth controlling for child's gender and age in years, mother's age at birth of the child, mother's educational level, location of the household (rural/urban), and survey-year and country-fixed effects. Observation weights are rescaled using each country's female population aged 15 to 49 years in the last year of the considered time period. ARI – acute respiratory infections.

Panel C. Predicted prevalence of fever across wealth quartiles over time, disaggregated by gender.

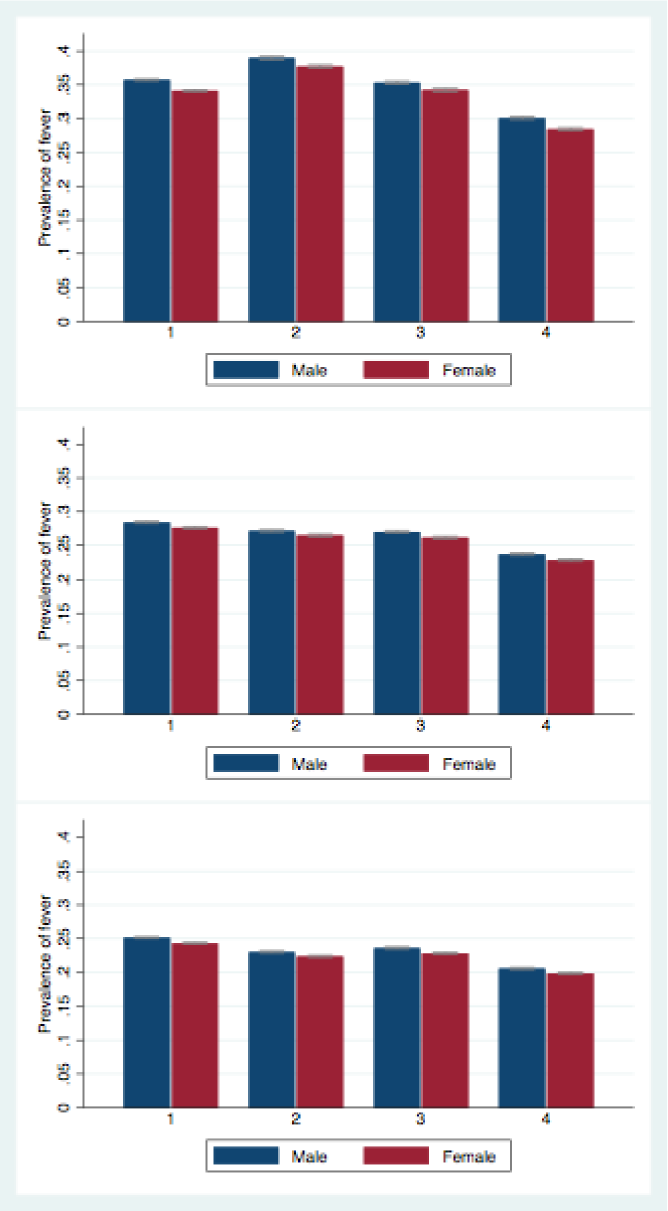

Note: Predicted prevalence of fever and 95% confidence intervals across wealth quartiles for all countries among boys and girls in three time periods: 1995-2001, 2002-2009, 2010-2017. Predicted prevalence is calculated separately for each time period from a regression of disease on wealth controlling for child's gender and age in years, mother's age at birth of the child, mother's educational level, location of the household (rural/urban), and survey-year and country-fixed effects. Observation weights are rescaled using each country's female population aged 15 to 49 years in the last year of the considered time period.

Panel D. Predicted prevalence of malaria across wealth quartiles, disaggregated by gender.

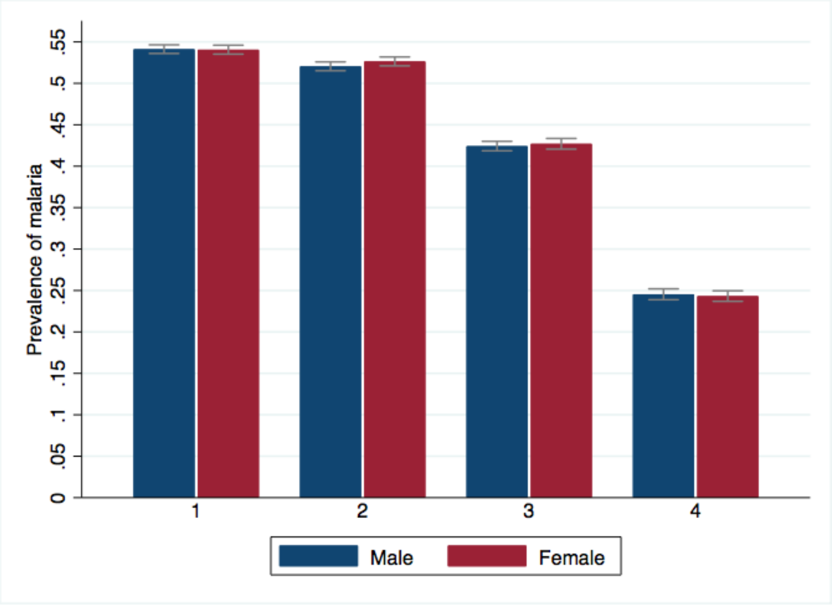

Note: Predicted prevalence of malaria and 95% confidence intervals across wealth quartiles for all countries among boys and girls, 2010-2017. Predicted prevalence is calculated from a regression of disease on wealth controlling for child's gender and age in years, mother's age at birth of the child, mother's educational level, location of the household (rural/urban), and survey-year and country-fixed effects. Observation weights are rescaled using each country's female population aged 15 to 49 years in 2017.
